# Phytohormonal Regulation Through Protein S-Nitrosylation Under Stress

**DOI:** 10.3389/fpls.2022.865542

**Published:** 2022-03-24

**Authors:** Anjali Pande, Bong Gyu Mun, Waqas Rahim, Murtaza Khan, Da Sol Lee, Geun Mo Lee, Tiba Nazar Ibrahim Al Azzawi, Adil Hussain, Chang Kil Kim, Byung Wook Yun

**Affiliations:** ^1^Laboratory of Plant Molecular Pathology and Functional Genomics, Department of Plant Biosciences, School of Applied Biosciences, College of Agriculture and Life Science, Kyungpook National University, Daegu, South Korea; ^2^Laboratory of Cell Biology, Department of Entomology, Abdul Wali Khan University, Mardan, Pakistan; ^3^Department of Horticultural Sciences, Kyungpook National University, Daegu, South Korea

**Keywords:** nitric oxide, phytohormones, S-nitrosylation, plant stress, proteins

## Abstract

The liaison between Nitric oxide (NO) and phytohormones regulates a myriad of physiological processes at the cellular level. The interaction between NO and phytohormones is mainly influenced by NO-mediated post-translational modifications (PTMs) under basal as well as induced conditions. Protein S-nitrosylation is the most prominent and widely studied PTM among others. It is the selective but reversible redox-based covalent addition of a NO moiety to the sulfhydryl group of cysteine (Cys) molecule(s) on a target protein to form S-nitrosothiols. This process may involve either direct S-nitrosylation or indirect S-nitrosylation followed by transfer of NO group from one thiol to another (transnitrosylation). During S-nitrosylation, NO can directly target Cys residue (s) of key genes involved in hormone signaling thereby regulating their function. The phytohormones regulated by NO in this manner includes abscisic acid, auxin, gibberellic acid, cytokinin, ethylene, salicylic acid, jasmonic acid, brassinosteroid, and strigolactone during various metabolic and physiological conditions and environmental stress responses. S-nitrosylation of key proteins involved in the phytohormonal network occurs during their synthesis, degradation, or signaling roles depending upon the response required to maintain cellular homeostasis. This review presents the interaction between NO and phytohormones and the role of the canonical NO-mediated post-translational modification particularly, S-nitrosylation of key proteins involved in the phytohormonal networks under biotic and abiotic stresses.

## Introduction

Nitric oxide (NO) is a multi-tasked, gaseous signaling molecule. This is due to its smallest diatomic molecular stucture which makes it highly diffusible across biomembranes. It is a key signaling molecule involved in numerous biotic and abiotic stress responses in plants (Yu et al., 2012). In plants, it regulates a plethora of physiological processes ranging from seed germination to plant growth, reproduction (Kwon et al., 2012), and defense against biotic and abiotic stresses (Hussain et al., 2019; Khan et al., 2019; Nabi et al., 2019). The biological functions mediated by NO are mainly chemical reactions of different nitrogenous products known as reactive nitrogen species (RNS) which includes NO, nitroxyl (HNO/NO^–^), nitrosonium cation (NO^+^), peroxynitrite (ONOO^–^), S-nitrosothiols (RSNOs), and dinitrosyl iron complexes. The transformation of NO into other redox forms under physiological conditions, is due to the susceptibility of free NO radical to oxidation and reduction. One of the key mechanisms by which NO mediates various pathways is through post-translational modification of various proteins. Among these, protein S-nitrosylation is perhaps the most prominent. It is the selective but reversible redox-based covalent addition of a NO moiety to the sulfhydryl group of cysteine (Cys) molecule(s) on a target protein to form S-nitrosothiols. The role of NO in signal transduction was first established in the animal system wherein the smooth vascular muscle cells it was found to bind to the heme group of guanylate cyclase (Murad, 1986) and consequent protein kinase-mediated, cyclic guanosine monophosphate (cGMP-dependent activation of potassium channels) (Bolotina et al., 1994). In plants, S-nitrosylation regulates a myriad of pathophysiological and physiological processes as well as those involved in biotic and abiotic stresses. NO interacts with all the major phytohormones including abscisic acid, auxin, gibberellic acid, cytokinin, ethylene, salicylic acid, jasmonic acid, brassinosteroid, and strigolactone (Asgher et al., 2017) during various metabolic and physiological conditions and environmental stress responses. S-nitrosylation of key proteins involved in the phytohormonal network occurs during their synthesis, degradation, or signaling roles depending upon the response required to maintain cellular homeostasis. This review presents NO-mediated S-nitrosylation of key proteins involved in phytohormonal networks under biotic and abiotic stresses.

## An Overview of Nitric Oxide-Mediated S-Nitrosylation, Transnitrosylation, and Denitrosylation

**FIGURE 1:**
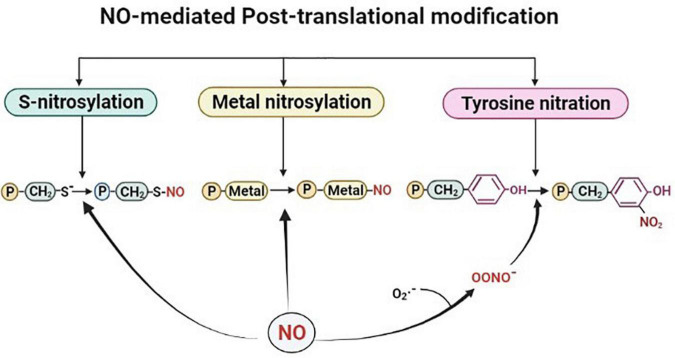
Outline of the nitric oxide (NO)-mediated post-translational modifications. Proteins are represented with letter “P.” Figure made in BioRender.com.

Post-translational modification by NO takes place by one of the three modification processes *viz*, S-nitrosylation (the formation of a nitrosothiol group on cysteine residues of target proteins), metal nitrosylation (interaction of NO with metalloproteins), and tyrosine nitration (covalent addition of NO to the tyrosine residues (as shown in Figure 1). Out of these three modifications, S-nitrosylation, also known as S-nitrosation plays crucial roles in various physiological and pathological processes by modulating protein activities. It is a highly conserved post-translational modification and is broadly studied and described in plants (Astier and Lindermayr, 2012). This dynamic and reversible process involves nitrosylation, transnitrosylation, and denitrosylation (Benhar et al., 2009; Astier and Lindermayr, 2012; Feng et al., 2019). S-nitrosylation or the more biochemistry-oriented term S-nitrosation is considered a non-enzymatic process, in which NO mediates the formation of S-nitrosothiols either directly or indirectly by higher nitrogen oxides (NO_*x*_), metal-NO intermediates, SNOs, or ONOO^–^ (Wang et al., 2006). S-nitrosylation of cysteine residues in the tripeptide glutathione (GSH) leads to the formation of low molecular weight SNOs which in turn function as NO donors depending on their redox potential under physiological conditions (Hess et al., 2005). This transfer of NO group from one thiol to another has also been reported and termed transnitrosylation and is known to be an important enzymatic process (Wu et al., 2010; Qu et al., 2011; Choi, 2018; Feng et al., 2019). In this process, the donor protein that carries and transfers the NO moiety to its target is termed as a transnitrosylase (Stomberski et al., 2019; Chen et al., 2020). A major determinant for NO transfer is the difference between the redox potential of the cysteine residues of the interacting proteins (donor protein-SNO and target protein with a free thiol). The process of transnitrosylation involves overturning of the first SNO-mediated regulation of the donor protein and may also be termed as denitrosylation (Choi, 2018; Feng et al., 2019). Denitrosylation of target proteins is promoted enzymatically and non-enzymatically which tightly regulates cysteine modification (Benhar et al., 2008, 2009; Astier et al., 2011). Transnitrosylation is carried out by proteins, also known as transnitrosylases that carry and transfer the NO group to its target (Anand and Stamler, 2012; Stomberski et al., 2019). In bacterial and mammalian cells, several transnitrosylases like cytochrome c, cytoglobin, caspase 3, thioredoxin, and hemoglobins have been identified and functionally characterized (Anand and Stamler, 2012). However, in the case of plants, GSNO is considered to possess major transnitrosylase activity, modulating the total SNO content (Wang et al., 2006; Yu et al., 2012). Studies have reported that the transnitrosylation of a protein-SNO to another protein with free thiol plays role in the regulation of NO-mediated regulatory mechanisms of signaling pathways (Nakamura et al., 2010; Choi et al., 2014). For instance, selective denitrosylation of S-nitrosylated proteins by Trx-h3 and Trx-h5 were found to regulate plant immunity in Arabidopsis (Kneeshaw et al., 2014). In another study, S-nitrosylation/denitrosylation are reported to be influenced by auxins in Arabidopsis roots. Overall, NO-mediated S-nitrosylation is a crucial signaling mechanism. However, regulators of similar processes like transnitrosylation and denitrosylation can also modulate protein functionality in response to environmental stress in plants.

## S-Nitrosylation of Target Proteins Involved in Phytohormonal Network

**FIGURE 2:**
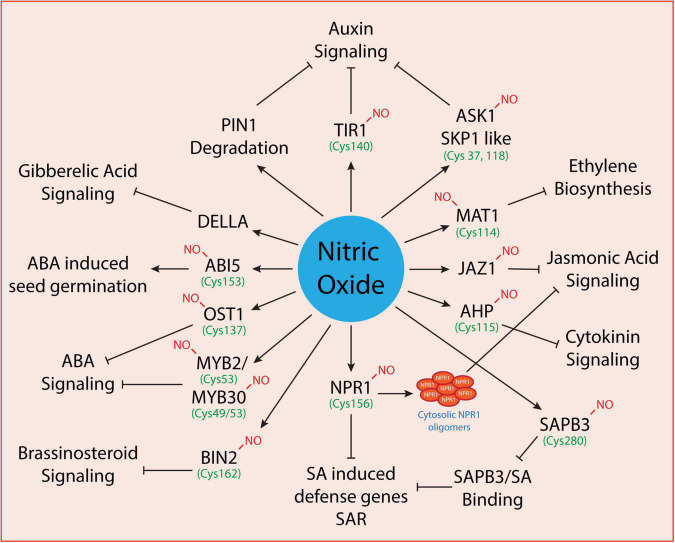
Regulation of phytohormonal network by nitric oxide. Some of the known proteins involved in phytohormonal signaling that are directly modulated by nitric oxide have been shown here. However, only a few are known to undergo S-nitrosylation (where the cysteine residues have been indicted with green color) while others need to be explored further.

Plants synthesize and maintain delicate levels of phytohormones to promote normal growth in plants under optimal conditions. However, under changing environmental conditions or upon exposure to biotic/abiotic stress conditions, phytohormones also regulate plant adaptation and survival by controlling the production of various stress-responsive proteins, antioxidants, and ion transporters. The phytohormones that promote the growth and govern the plant survival under stressful conditions include auxins, gibberellins, jasmonic acid, salicylic acid, abscisic acid, cytokinins, ethylene, brassinosteroids, and strigolactones. These undergo coordinated interactions with various signaling molecules like nitric oxide and hydrogen peroxide to regulate their activity depending on the severity of the environmental stress. Largely, the liaison between NO and phytohormones regulates a myriad of physiological processes (as shown in Figure 2) at the cellular level. Under environmental stress conditions, NO and phytohormonal coordination regulates gene express and activities of antioxidative enzymes. The phytohormonal levels are regulated either at the biosynthesis, distribution, or signaling stage. NO can regulate the level of hormones at transcriptional (Bethke et al., 2007; Lozano-Juste and León, 2011) or post-translational levels (Lindermayr et al., 2005; Terrile et al., 2012; Feng et al., 2019). S-nitrosylation is a common signaling mechanism mediated by nitric oxide which regulates hormonal signaling at the post-translational level. Thus, S-nitrosylated proteins involved in phytohormonal activity are regulated at the pre-receptor level (as in the case of ethylene), receptor level (as in the case of auxin signaling where TIR1 binds auxin directly conferring an increased affinity for AUX/IAA proteins) and post-receptor levels (as in the case of cytokinin and abscisic acid signaling where hormonal binding to the receptor determines the response) of phytohormonal activity. Overall, we discuss below the interplay between NO and phytohormones and the S-nitrosylation of target proteins involved in the phytohormonal network at various levels.

## S-Nitrosylated Protein in Auxin and Gibberellin Signaling

The phytohormone, auxin plays a crucial role in various developmental processes both under normal and stress conditions. The major function of auxin is the formation, development, and maintenance of roots (Overvoorde et al., 2010). However, for the efficient regulation of plant development under changing environmental conditions auxins interact with other phytohormones and signaling molecules like NO (Freschi, 2013). The NO-auxin interplay suggests an increase in NO after auxin is applied to the roots or in auxin overexpressing mutant lines which lead to the speculations that NO may act downstream of auxins (Correa-Aragunde et al., 2016). The interaction between NO and auxin has been studied under heavy metal stress conditions with the application of NO-donors (mostly sodium nitroprusside). However, the results have been contradictory suggesting that NO accumulation is responsible for an increase in auxin-dependent root elongation to overcome mercury-induced toxicity in rice (Chen et al., 2015) whereas it inhibits root meristem growth by repressing auxin signaling under cadmium stress in Arabidopsis (Yuan and Huang, 2016).

**FIGURE 3:**
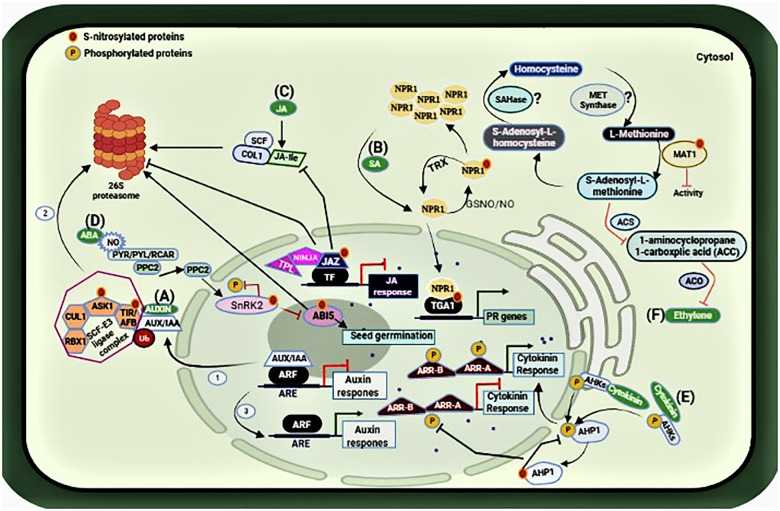
Representation of the S-nitrosylated proteins involved in the hormonal network. **(A)** S-nitrosylation of ASK1 and TIR1 lead to the proteolytic degradation of AUX/IAA, thus initiating auxin response (steps 1–3). **(B)** S-nitrosylation/glutathionylation of NPR1leads to its oligomerization while S-nitrosylation of TGA1 promotes its interaction with NPR1 enabling the expression of PR genes. **(C)** S-nitrosylation prevents the interaction between JAZ1 repressor proteins and COI1, which is a subunit of the SCF ubiquitin E3 ligase complex for its proteasomal degradation. Thus, allowing JAZ1 to recruit its co-repressors NINJA and TPL to repress JA signaling. **(D)** S-nitrosylation of ABI5 also leads to its proteasomal degradation, thus promoting seed germination in the presence of NO. **(E)** S-nitrosylation of AHP1 inhibits its phosphorylation, compromising cytokinin response. **(F)** S-nitrosylation of MAT1 suppresses the activity of 1-aminocyclopropane1-carboxplic acid (ACC) synthesis and ACC oxidase, thereby affecting the synthesis of ethylene Moreover, S-nitrosylation of SAHase and MET synthase are still under investigation (shown with question marks). Figure made in BioRender.com.

Auxin signaling response is suppressed due to the dimerization of AUXIN/INDOLEACETIC ACID (AUX/IAA) transcription factor with AUXIN RESPONSE FACTOR (ARF) transcriptional activators that are present on the auxin-responsive promoter elements (ARE). However, in the presence of auxin, this transcription factor is subjected to ubiquitin (Ub)-mediated proteasomal degradation with the help of SCF-E3 ligase complex (steps 1–3), and auxin response initiates (Ramos Báez and Nemhauser, 2021). The SCF-E3 complex comprises of four subunits: CULLIN1 (CUL1), S-PHASE KINASE-ASSOCIATED PROTEIN 1-LIKE1 (ASK1), RING BOX 1 (RBX1), and TRANSPORT INHIBITOR RESISTANT 1 (TIR1)/AUXIN SIGNALING F-BOX(AFB). S-nitrosylation of TIR1 and ASK1 in this pathway enhances the protein-protein interaction modulating the SCF^*TIR*1/AFBs^ complex assembly (Iglesias et al., 2018), and hence auxin signaling response in plants, as shown in Figure 3A.

Independent studies on the cross talk between NO and auxin in controlling plant development (probably through S-nitrosylation) has been reported by Fernández-Marcos et al. (2011) and Terrile et al. (2012). In Fernández-Marcos et al. (2011) reported that NO causes root apical meristem defects and growth inhibition in Arabidopsis by enhancing the degradation of the auxin efflux transporter PIN-FORMED 1 (PIN1), thereby reducing PIN1-dependent acropetal auxin transport. Later, Terrile et al. (2012) showed that NO also enhances the interaction between the auxin receptor TIR1 and Aux/IAA suppressor. NO directly targets the auxin receptor TIR1 for S-nitrosylation of its Cys_140_ residue which is critical for TIR1 function and its interaction with AUX/IAA repressor.

Nitric oxide also impacts auxin signaling pathway through SCF^*TIR*1/AFB^ E3 ubiquitin ligase complex assembly *via* S-nitrosylation of the ARABIDOPSIS SKP1-LIKE1 (ASK1) at Cys_37_ and Cys_118_. S-nitrosylation of ASK1 influences its binding with TIR1/AFB2 and Cullin1 (CUL1) which in turn promotes SCF^*TIR*1/^ and SCF^*AFB*2^ assembly, resulting in an impaired auxin signaling activation (Iglesias et al., 2018).

Furthermore, an interesting study by Yang et al. (2015) reported that S-Nitrosylation of the NO scavenger ascorbate peroxidase (APX1) at Cys_32_ positively regulates its activity during stress. However, auxin induces the de-nitrosylation and partial inhibition of APX1 (Correa-Aragunde et al., 2013). This indicates the presence of an auxin-mediated APX1 S-nitrosylation/de-nitrosylation equilibrium at the cellular level that contributes to a fine-tuned control of reactive oxygen species (Fares et al., 2011).

Similarly, gibberellin constitutes a large family of tetracyclic diterpenoid phytohormones regulating plant growth and development. Nitric oxide regulates DELLA content and PIF expression to promote photomorphogenesis in *Arabidopsis* (Lozano-Juste and León, 2011). NO negatively regulates gibberellin signaling by promoting the accumulation of the gibberellin signaling repressor DELLA and decreasing the expression of phytochrome-interacting factors (PIFs) (Richter et al., 2010). This is further supported by studies involving the Arabidopsis loss of function mutant lines *nox1* and *gsnor1-3* that accumulate more NO/SNOs, and have abnormal auxin and gibberellin responses (Fernández-Marcos et al., 2011; Terrile et al., 2012). However, no S-nitrosylated target protein have been reported so far in the case of gibberellic acid.

## S-Nitrosylated Proteins in Salicylic Acid and Jasmonic Acid Signaling

Salicylic Acid (SA), and Jasmonic Acid (JA) are key phytohormones that regulate plant responses to infection by a variety of pathogens including fungi, bacteria, viruses, and others (Fujita et al., 2006; Loake and Grant, 2007). NO exerts its role on these pathways to regulate plant defense during infection. Salicylic acid regulates plant responses to infection by pathogens and is essential for the establishment of resistance mechanisms such as host cell death and systemic acquired resistance (SAR). The S-nitrosoglutathione reductase (GSNOR) is a key enzyme regulating cellular S-nitrosothiol levels *via* denitrosylation (Malik et al., 2011). The Arabidopsis *atgsnor1-3* line has significantly higher levels of cellular SNOs and perturbed SA biosynthesis and signaling. Tada et al. (2008) showed that plant immunity requires conformational changes of NPR1 *via* S-nitrosylation. NPR1, a master regulator of SA-mediated plant defense is sequestered in the cytoplasm as an oligomer. Upon infection, it monomerizes and is translocated to the nucleus to activate a battery of *pathogenesis-related* (*PR*) genes (Kinkema et al., 2000). However, in unchallenged plants, the oligomer to monomer switch is regulated by S-nitrosylation at Cys_156_ inhibiting its monomerization (Tada et al., 2008). Furthermore, NO accretion during the nitrosative burst after infection promotes S-nitrosylation of the *Arabidopsis thaliana* salicylic acid-binding protein 3 (ATSABP3) at Cys_280_ which not only suppresses its binding to SA but also inhibits its carbonic anhydrase activity (Wang et al., 2009). On the other hand, S-nitrosylation also regulates SAR by targeting the NPR1/TGA1 system. As described above, SA induces thioredoxin (TRX) which facilitates denitrosylation of NPR1 for its monomerization during plant immune response (Kneeshaw et al., 2014) and thus facilitating its translocation to the nucleus where they interact with the basic leucine zipper transcription factor TGA to promote TGA attachment to the promoters of PR genes (Tada et al., 2008). Notably, it has been demonstrated that S-nitrosylation (and S-glutathionylation) improves TGA1 binding to the PR1 promoter (Lindermayr et al., 2010) as shown in Figure 3B. Interestingly, the cytosolic NPR1 also contributes to the suppression of the JA pathway. Hence, it can be concluded that distinct redox signals work to maintain a cellular redox balance to regulate plant immunity and that S-nitrosylation is a key mediator of integrated phytohormonal networks concerning plant immunity.

Jasmonic acid (JA) is an important phytohormone involved in plant development and response to injury, attack by insects, and necrotrophic pathogens. Jasmonic acid (JA) signaling depends on the interaction between JASMONATE ZIM DOMAIN (JAZ1) repressor proteins and CORONATINE-INSENSITIVE 1 (COI1), which is a subunit of the SCF ubiquitin E3 ligase complex for its proteasomal degradation (Ghorbel et al., 2021). Ayyar (2016) investigated the relationship between NO and JA signaling during the plant immune response. Increasing NO content appeared to have a negative effect on JA signaling as the NO over-accumulating *atgsnor1-3* line compromised JA signaling. Her results from the biotin switch assay indicated that JAZ1 is S-nitrosylated at CyS_229_ *in vitro*. Further investigations involving Flag-tagged JAZ1 over-expression in the *atgsnor1-3* line also indicated the S-nitrosylation of JAZ1 *in vivo*. This implies that JA signaling during insect attack or infection by necrotrophs such as *Botrytis cinerea* is under redox control. She proposed that JAZ1 S-nitrosylation may block its interaction with the JA receptor component COI1, while enabling JAZ1 to recruit its co-repressors NINJA (Novel INteractor of JAZ) and TPL (TOPLESS) to inhibit turnover by the proteasome, consequently enabling prolonged JAZ1-mediated suppression of JA signaling, as shown in Figure 3C.

## S-Nitrosylated Proteins in Abscisic Acid Signaling

The isoprenoid phytohormone Abscisic acid (ABA) regulates a variety of physiological processes in plants including stomatal movement, protein storage, and adaptation to abiotic stresses such as cold, drought, and salt stresses. At the base of these events are complex signaling networks involving multiple components such as K^+^, Ca^2+^, MAP kinases (MAPK), H_2_O_2_, and others (Fan et al., 2004). NO is also known to be involved in various stress responses by regulating these components (Xu et al., 2015; Montilla-Bascón et al., 2017; Nabi et al., 2019; Lau et al., 2021). Ca^2+^ regulates stomatal movement during drought stress (Zou et al., 2010). On the other hand, NO exerts its effect on Ca^2+^ *via* S-nitrosylation of Ca^2+^ channels and transporters. Foresi et al. (2015) showed that NO triggers stomatal closure *via* regulation of the Ca^2+^- sensitive Cl^–^ and K^+^ channels at the plasma membrane of the guard cells. However, some studies have also demonstrated a synergistic relationship between NO and Ca^2+^ in response to drought stress (Niu et al., 2017; Silveira et al., 2020). S-nitrosylation of transcription factors like the basic leucine zipper transcription factor ABI5, MYB2, and MYB30 is involved in abscisic acid (ABA) mediated regulation under drought stress.

The basic leucine zipper transcription factor ABI5 (ABA-INDUCED 5) regulates ABA-mediated seed germination and early seedling growth and is considered as a NO sensor, as Albertos et al. (2015) showed that NO regulates ABI5 at both transcription and translational levels. ABA signaling in the presence of NO involves tyrosine nitration of the PYRABACTIN RESISTANCE-LIKE REGULATORY COMPONENTS (PYR/PYL/RCAR) which inhibits its interaction with ABA thereby enabling the activity of type 2C protein phosphatases (PPC2), which inactivates SUCROSE NON-FERMENTING1 (SNF1)-RELATED PROTEIN KINASE2.6 (SnRK2.6) by dephosphorylation, which lead to the inhibition of ABI5. Moreover, S-nitrosylation of ABI5 at Cys_153_ facilitates ABI5 degradation by enhancing its interaction with the E3 ligase complex promoting seed germination and seedling growth (Albertos et al., 2015), as shown in Figure 3D. However, another study in the same year by Wang et al. (2015) reported that NO negatively regulates ABA signaling through S-nitrosylation of the open stomata 1 (OST1) at Cys_137_ in the guard cells. OST1 is a sucrose non-fermenting 1 (SNF1)-related protein kinase 2.6 (SnRK2.6). Loss of GSNOR1 function in the Arabidopsis *atgsnor1-3* plants results in NO overaccumulation in the guard cells, leading to S-nitrosylation of SnRK2.6 abolishing ABA-dependent stomatal closure.

Transcription factors play an important role in regulating the expression of key genes involved in important physiological processes. The expression of the MYB2 transcription factor increases in response to ABA and water stress as Arabidopsis plants over-expressing MYB2 are ABA-hypersensitive indicating that MYB2 may be involved in ABA singling. On the other hand, MYB30 is involved in the hypersensitive response (HR) and is characterized by a vast production of ROS and NO. Both MYB2 and MYB30 have been shown to undergo S-nitrosylation at Cys_53_ and Cys_49/53_, respectively, which abolishes their DNA binding ability (Serpa et al., 2007; Tavares et al., 2014). Taken together, it can be concluded that both ABA and NO are key signaling molecules that regulate responses to drought stress.

## S-Nitrosylated Proteins in Cytokinin and Ethylene Signaling

In plants, cytokinin is an essential phytohormone regulating plant growth and development. As Feng et al. (2013) describe, investigations indicate cytokinin promotes phosphorelay activity through the membrane-bound hybrid HISTIDINE PROTEIN KINASES (AHKs) to HISTIDINE PHOSPHOTRANSFER PROTEIN 1 (AHP1) and then to primary RESPONSE REGULATOR TYPE B (ARR-B) and primary RESPONSE REGULATOR TYPE A (ARR-A) to promote cytokinin signaling response. They showed that NO negatively regulates cytokinin signaling by inhibiting the phosphorelay system *via* S-nitrosylation of AHP1 at Cys_115_ rendering it unable to phosphorylate, as shown in Figure 3E. They also showed that a non-nitrosylatable mutant protein AHP1 partially relieves the negative effects of NO on cytokinin signaling. Their findings illustrate that cytokinin signaling and redox signaling *via* S-nitrosylation coordinated plant growth and development.

Similarly, ethylene is a versatile phytohormone that regulates both growth/development and senescence. Ethylene biosynthesis is regulated by NO through S-nitrosylation of enzymes involved in the Yang cycle (or the methyl-methionine cycle) (Pattyn et al., 2021). The activity of 1-aminocyclopropane1-carboxplic acid (ACC) synthesis, as well as ACC oxidase, is suppressed due to S-nitrosylation of METHIONINE ADENOSYLTRANSFERASE (MAT1) thereby affecting the synthesis of ethylene through ACC synthase (ACS) and (ACC) oxidase (ACO) (Lindermayr et al., 2006). Moreover, S-nitrosylation of S-adenosylhomocysteinase (SAHase) and cobalamin-independent methionine synthase (MET synthase) are still under investigation. Lindermayr et al. (2006) reported the inactivation of METHIONINE ADENOSYLTRANSFERASE (MAT1) due to its S-nitrosylation at Cys_114_. In addition, enzymes such as S-adenosylhomocysteinase and cobalamin-independent methionine synthase are also a part of the ethylene pathway and have been found to be the targets of S-nitrosylation by NO (Lindermayr et al., 2005; Abat et al., 2008). This indicates a multi-step control of ET biosynthesis and signaling in plants *via* S-nitrosylation as shown in Figure 3F. Table 1 below indicates the target proteins involved in phytohormonal network.

**TABLE 1:** S-nitrosylation of target proteins involved in phytohormonal network.

S.NO.	Phytohormone	Target protein for S-nitrosylation	Studied plants	Stress responses	References
1.	Auxin	TIR1	*A. thaliana*, *Triticum aestivum* L.	Facilitate protein-protein interaction. Negatively regulates basal defense against fungi.	Terrile et al., 2012; Fousia et al., 2018; Su et al., 2021
		ASK1	*A. thaliana*	Enhances binding to CUL1 and TIR1/AFB2. Activates SAR against *P. syringae pv. maculicola*.	Iglesias et al., 2018; Zhou et al., 2021
2.	Abscisic acid	MYB2	*A. thaliana*, *Scutellaria*	Inhibition of protein activity. Response to abiotic stresses.	Baek et al., 2013a,b; Jia et al., 2020
		MYB30	*A. thaliana*	Inhibition of protein activity. Response to biotic and abiotic stresses.	Liao et al., 2017; Mabuchi et al., 2018; Gong et al., 2020
		ABI5	*A. thaliana*	Degradation of protein. Response to abiotic stresses.	Wang et al., 2015; Skubacz et al., 2016
		SnRK2.2 and SnRK2.3	*A. thaliana, Zea Mays*	Inactivation of proteins. Response to abiotic stresses.	Fujii and Zhu, 2009; Nakashima et al., 2009
		SnRK2.6/OST1	*A. thaliana*	Inhibition of proteins. Differential response to abiotic stresses.	Yoshida et al., 2002; Tada et al., 2008; Wang et al., 2015
3.	Cytokinin	AHP1	*A. thaliana*	Inhibition of protein activity. Regulates responses to both biotic and abiotic factors.	Feng et al., 2013; Cortleven et al., 2019
4.	Salicylic acid	NPR1	*A. thaliana*, *Oryza sativa*, *Triticum aestivum* L.	Conformational changes in protein. Regulates resistance to a wide range of pathogens.	Lindermayr et al., 2010; Molla et al., 2016; Backer et al., 2019
		TGA1	*A. thaliana*	Facilitate NPR1-TGA1 interaction. Regulates resistance against biotic stress.	Shearer et al., 2012; Backer et al., 2019
		SABP3	*A. thaliana*, *Nicotiana tabacum*	Immune response activation. SA and carbonic anhydrase (CA) activity.	Wang et al., 2009
5.	Jasmonic acid	JAZ1	*A. thaliana*	Suppression of protein activity. Regulates responses to both biotic and abiotic factors.	Ayyar, 2016
6.	Ethylene	MAT1	*A. thaliana*	Inhibition of protein function. Regulates responses to both biotic and abiotic factors.	Lindermayr et al., 2005; Khan et al., 2017
7.	Brassinosteroid	BIN2	*Zea mays*	Interference with structural assembly. Oxidative stress tolerance.	Mao and Li, 2020
8.	Strigolactones	MAX4/CCD8 and MAX2 D53 and D5 (predicted)	*A. thaliana*, *Oryza sativa*	Involved in biosynthesis. Strigolactone signaling.	Kolbert, 2019

## S-Nitrosylated Proteins Predicted in Brassinosteroid and Strigolactone Signaling

Brassinosteroids are a class of phytohormones that are polyhydroxylated and steroidal similar to the steroid hormones in animals. These steroidal hormones regulate a wide variety of physiological plant processes including growth, development, immunity, and stress responses (Ahammed et al., 2020; Hussain et al., 2020; Nolan et al., 2020; Ortiz-Morea et al., 2020). Brassinosteroid signaling involves a constitutively active kinase, BIN2 (Brassinosteroid-Insensitive2) which is suggested to be s-nitrosylated at the conserved Cys_162_ site as indicted by an *in vitro* assay (Mao and Li, 2020). It has been further suggested in this study that this modification could interfere with its structural assembly or its interaction with the two key transcription factors, BES1 (bri1-EMS suppressor1) and BZR1 (Brassinazole-Resistant1). However, to confirm these observations further *in vivo* investigations are required.

Strigolactones belong to a more recently studied group of phytohormones involved in growth and development processes mainly in symbiotic mycorrhizal plant-fungi association (Faizan et al., 2020). These are also involved in the regulation of stress responses in plants. *In silico* analysis of NO-mediated post-translational modification of proteins involved in strigolactones biosynthesis and signaling in *A. thaliana* and *Oryza sativa* predicted S-nitrosylation of MAX4/CCD8 (carotenoid cleavage dioxygenases) in Arabidopsis involved in biosynthesis while MAX2 (MORE AXILLARY GROWTH2 in Arabidopsis), and D53 (Dwarf 53) and D5 (Dwarf 5) in *O. sativa* involved in strigolactone signaling pathway (Kolbert, 2019). However, detailed studies are required to confirm such predictions *in vitro* and *in vivo*.

## Conclusion and Future Prospects

Plants being sessile, are vulnerable to various environmental changes, challenging them to adjust and grow under such adversities. Plants can survive and grow under these challenging situations due to the coordinated signaling mechanisms involving phytohormones and other stress-responsive molecules like NO and H_2_O_2_. NO is an essential signaling molecule in signaling cascade interaction with almost all phytohormones. During the past few years, there is an extensive quest for search to explore the multiple and widely diverse mechanisms regarding plant hormones and NO interaction. Undoubtedly. NO has a dual role in the up and downregulation of plant hormones. The NO-phytohormonal interaction is mostly through NO-mediated post-translational modifications regulating the synthesis, distribution, degradation, and conjugation of the elements involved in the plant hormonal transport and signaling. However, there are still studies lagging in explaining how NO interacts with hormones and hormones-related proteins at translational and posttranslational levels. Moreover, the interplay among NO and phytohormones needs to be explored in future work. Overall, it requires extensive research and investigation to explore the coordination between NO and various phytohormonal signaling cascades operating in plants under the pressure of multiple stresses.

## Author Contributions

AP contributed to the writing—original draft preparation and images. BM contributed to the review, editing and gathering resources. WR contributed to the preparation of the table and its contents. MK and TA contributed to the section on S-nitrosylation of target proteins involved in phytohormonal network. DL and GL contributed to the illustration and visualization of the images. AH revised and improved the manuscript and images. CK critically discussed and revised the manuscript. BY revised the final draft and supervised the entire manuscript. All authors contributed to the article and approved the submitted version.

## Conflict of Interest

The authors declare that the research was conducted in the absence of any commercial or financial relationships that could be construed as a potential conflict of interest.

## Publisher’s Note

All claims expressed in this article are solely those of the authors and do not necessarily represent those of their affiliated organizations, or those of the publisher, the editors and the reviewers. Any product that may be evaluated in this article, or claim that may be made by its manufacturer, is not guaranteed or endorsed by the publisher.
